# Robotic Surgical Systems in Oral and Maxillofacial Surgery: A Systematic Review and Meta-Analysis

**DOI:** 10.4317/jced.63415

**Published:** 2025-12-30

**Authors:** Sergey Y. Ivanov, Alexander A. Muraev, Sergey N. Filatov, Leonid L. Borozdkin, Artyom M. Gusarov, Darya Y. Milyukova, Sergey G. Ivashkevich, Sergey S. Ivanov

**Affiliations:** 1 Department of Oral and Maxillofacial Surgery and Surgical Dentistry, Peoples´Friendsfip University of Russia (RUND University), Moscow, Russia; 2 Department of Oral and Maxillofacial Surgery named after Academician N.N. Bazhanov, E.V. Borovsky Institute of Dentistry, I.M. Sechenov First Moscow State Medical University of the Ministry of Health of the Russian Federation (Sechenov University), Moscow, Russia; 3 Dental Hospital Zhongke Yagu China. Wenhua Road No.385 Rongyao Suhe F3, Kuiwen. Weifang Shandong China; Postal code: 261000

## Abstract

**Background:**

Relevance. In recent years, robotic surgical technologies have been firmly integrated into the practice of many surgical specialties. However, their adoption in dentistry and maxillofacial surgery remains limited. This is largely due to the unique anatomical features of the facial region and the exceptionally high demands for precision and equipment adaptability. At the same time, the growing interest in minimally invasive and highly accurate surgical interventions underscores the need for a scientific evaluation of the effectiveness of such technologies. Objective. This study aimed to systematically review the available clinical evidence on the use of robot-assisted surgical systems in oral and maxillofacial procedures, focusing on their accuracy, safety, and clinical feasibility.

**Material and Methods:**

The review was conducted in accordance with the PRISMA methodology. Studies involving patients who underwent implant or other maxillofacial surgical procedures using robotic systems were included. A systematic search was performed via the Cochrane Library platform, which simultaneously searches MEDLINE (PubMed) and Embase databases. The following keywords were used: (MeSH descriptor: [Robotics] explode all trees OR MeSH descriptor: [Robotic Surgical Procedures] explode all trees OR "robotic surgery":ti,ab,kw OR "robotic assisted":ti,ab,kw OR "robot":ti,ab,kw) AND ("maxillofacial surgery":ti,ab,kw OR "dental implant":ti,ab,kw OR "oral implantology":ti,ab,kw OR "oral surgery":ti,ab,kw). The level of evidence was assessed according to the GRADE, RoB 2.0, and ROBINS-I scales. Dual screening and data extraction were independently performed by two reviewers. The review protocol was prospectively registered in the PROSPERO database (CRD420251137197).

**Results:**

A total of 18 studies met the inclusion criteria. In nearly all studies, robotic systems demonstrated high implant placement accuracy (mean deviations less than 1 mm and 3°), substantially outperforming conventional techniques. The safety profile was consistently favorable. In transoral oncologic surgery, robotic systems showed comparable or superior functional outcomes. However, most studies were limited in sample size and follow-up duration, necessitating cautious interpretation of the results.

**Conclusions:**

Robot-assisted technologies in implantology and maxillofacial surgery have the potential to enhance precision and safety. Nevertheless, the overall certainty of evidence (GRADE) is rated as moderate to low. Larger-scale studies are required to confirm these findings.

## Introduction

Over the past two decades, robotic surgical systems have become firmly established in the operating practice of several surgical disciplines - urology, gynecology, general surgery, and thoracic surgery (1). Their advantages include enhanced precision of manipulations, improved visualization, and tremor reduction, all of which are particularly critical when operating in anatomically complex and confined spaces (2). One of the most promising directions for further development is the integration of robotic technologies into oral and maxillofacial surgery (OMFS), where the high demands for instrument positioning accuracy, minimal invasiveness, and favorable cosmetic outcomes make their use especially relevant (3 , 4). Despite significant technological progress, the adoption of robotics in oral and maxillofacial surgery has been comparatively delayed. This is attributed both to the anatomical and topographical complexity of the maxillofacial region and to the limited adaptability of existing robotic platforms for procedures performed in small operative fields with a high density of critical anatomical structures (5). Nonetheless, as robotic systems continue to evolve, interest in their application within dentistry and OMFS has been steadily increasing. However, robust scientific evidence of their clinical and cost-effectiveness is still required (6 , 7). Although the interest in robotic technologies in OMFS is growing, nearly half of all related studies remain at the preclinical stage, significantly limiting their clinical relevance (8). Existing publications are often limited to case reports, small retrospective series, or technical notes lacking standardized outcome assessment (9). Furthermore, there is a lack of validated classification of indications, as well as standardized parameters for evaluating the effectiveness and safety of robot-assisted interventions. These require further refinement before robotic systems can be fully integrated into clinical practice (10). Additionally, there is a noticeable shortage of comparative studies between robotic and conventional surgical techniques, which complicates the development of evidence-based clinical guidelines. Critically important aspects such as the cost-effectiveness of robotic systems, surgeons' learning curves, and platform-specific technical limitations are also rarely discussed in the literature (11). All of these factors highlight the necessity of a systematic analysis of the available data to objectively assess both the potential and the limitations of robotic systems in oral and maxillofacial surgery. The aim of this systematic review was to evaluate the efficacy and safety of robotic surgical systems in oral and maxillofacial surgery and dentistry. Following the PICO framework: Population - patients undergoing dental implantation or transoral oncologic surgery; Intervention - robot-assisted surgery; Comparators - conventional techniques and computer-assisted (non-robotic) implantation; Outcomes - procedural accuracy, complications, implant survival, and oncologic outcomes (resection margins, local tumor control, progression-free survival, and overall survival).

## Material and Methods

This review was conducted in accordance with the PRISMA (Preferred Reporting Items for Systematic Reviews and Meta-Analyses) guidelines. Its objective was to systematically summarize data on the use of robotic systems in patients with various pathologies of the oral and maxillofacial region. Inclusion and Exclusion Criteria Original studies meeting the following conditions met the inclusion criteria: Randomized controlled trials (RCTs), prospective, and retrospective studies; Adult and pediatric patients who underwent dental implant placement or robot-assisted procedures in the maxillofacial region (including oncologic surgeries, reconstructions, and implantology). Exclusion criteria: Case reports, technical notes, or descriptive case series with fewer than 10 patients; Literature reviews or letters to the editor; Studies published before 2015; Experimental studies conducted on animals or models. The review protocol was prospectively registered in the PROSPERO database (CRD420251137197). 2.3. Search Strategy A systematic literature search was conducted using the Cochrane Library platform, which enables simultaneous searching of PubMed/MEDLINE and Embase databases. Both controlled vocabulary terms (MeSH, Emtree) and free-text keywords were used, with the search limited to the period 2015-2025. The final search was performed on May 31, 2025. Search strategies were adapted for each database to include terms related to robotic surgery, dental implants, and placement accuracy. Only studies published in English were considered. Example of the search strategy in the Cochrane Library: #1 MeSH descriptor: [Robotics] explode all trees #2 MeSH descriptor: [Robotic Surgical Procedures] explode all trees #3 (robotic surgery):ti,ab,kw #4 (robotic assisted):ti,ab,kw #5 (robot):ti,ab,kw #6 (maxillofacial surgery):ti,ab,kw #7 (dental implant):ti,ab,kw #8 (oral implantology):ti,ab,kw #9 (oral surgery):ti,ab,kw #10 (#1 OR #2 OR #3 OR #4 OR #5) AND (#6 OR #7 OR #8 OR #9) Two independent reviewers performed title and abstract screening. Full-text articles were then assessed for eligibility according to inclusion criteria. Disagreements were resolved by consensus. Duplicate publications were removed at the initial screening stage. Outcomes and Effect Measures. Primary outcomes (implantation): coronal and apical deviations (mm), angular deviation (°). Secondary outcomes: depth deviation (mm), operative time (min), complication rate (%), and implant survival (6-12 months). For continuous outcomes, mean differences (MD) with 95% confidence intervals (CI) were calculated (negative MD = favors the robotic system). For dichotomous outcomes, risk ratios (RR) were used. Data Synthesis and Statistical Analysis. Separate pooled analyses were performed for the following comparisons: robot vs freehand, robot vs static CAIS, robot vs dynamic navigation; Pooling was conducted when k 2, and the final results indicated k (and N, when available). A random-effects model (DerSimonian-Laird) was applied, with heterogeneity assessed using the I² statistic. Statistical analyses were performed using SPSS, with = 0.05. Small-study effects were evaluated when k 10. Meta-Analysis Scope. A total of 18 studies met the inclusion criteria. Among them, 12 addressed dental implantation, and 10 comparative studies were included in the meta-analysis: robot vs static - k=5, robot vs dynamic - k=5, robot vs freehand - k=5. Studies related to TORS (Transoral Robotic Surgery) and other interventions were included only in the narrative synthesis. Risk of Bias Assessment The methodological quality of the included studies was assessed according to their design: RoB 2.0 (Cochrane Risk of Bias 2.0 tool) - for randomized controlled trials (RCTs); ROBINS-I (Risk of Bias in Non-Randomized Studies - of Interventions) - for non-randomized interventional studies. Each tool classified studies into three levels of risk: low, some concerns, or high risk of bias. Additionally, the GRADE approach was applied to determine the certainty of evidence (high, moderate, low, or very low). All assessments were independently performed by two reviewers, with disagreements resolved through discussion or by consulting a third expert.

## Results

**Figure 1:**
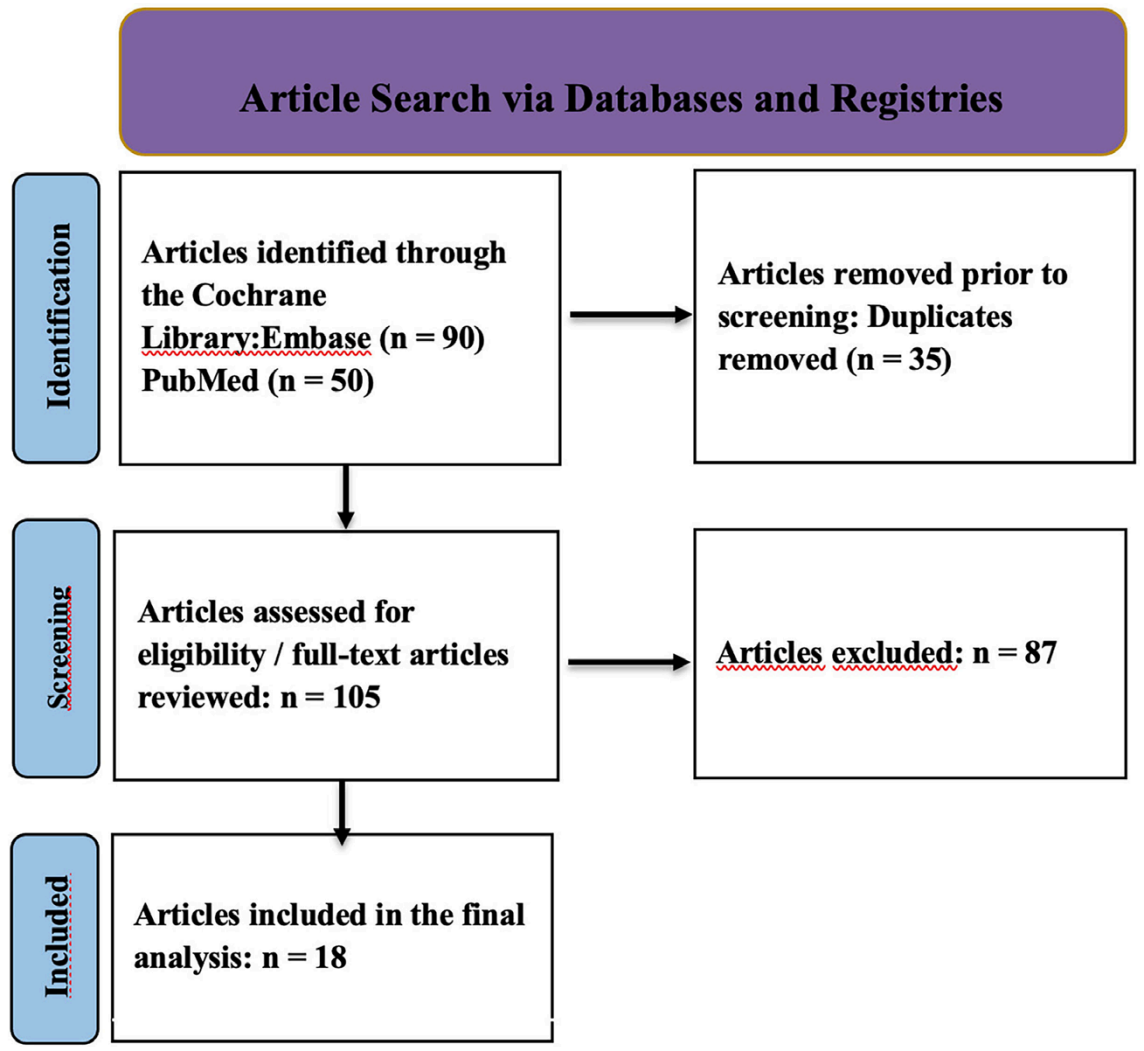
Prisma flow diagram.

The systematic search of PubMed and Embase databases initially identified 140 publications (50 from PubMed and 90 from Embase). After removing duplicates, 105 unique records remained. At the title and abstract screening stage, 87 records were excluded as irrelevant to the review's aims and criteria. Ultimately, 18 studies fully meeting the inclusion criteria were incorporated into the qualitative synthesis. These studies formed the basis for data aggregation and subsequent analysis of the efficacy and accuracy of robot-assisted interventions in dentistry and otorhinolaryngology (Fig. 1).

[caption id="attachment_1957" align="alignnone" width="300"]1 Screenshot[/caption]

**Figure 2:**
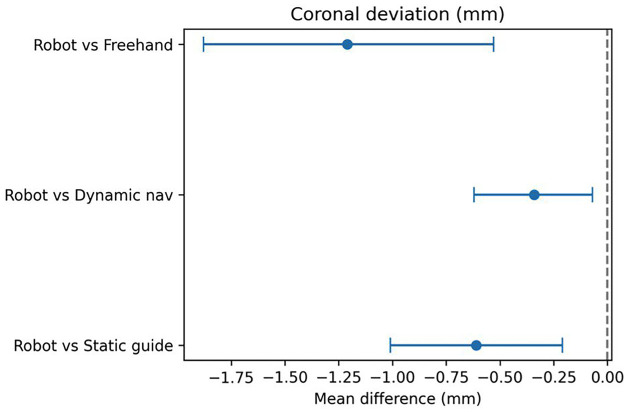
Coronal deviation (mm).

**Figure 3:**
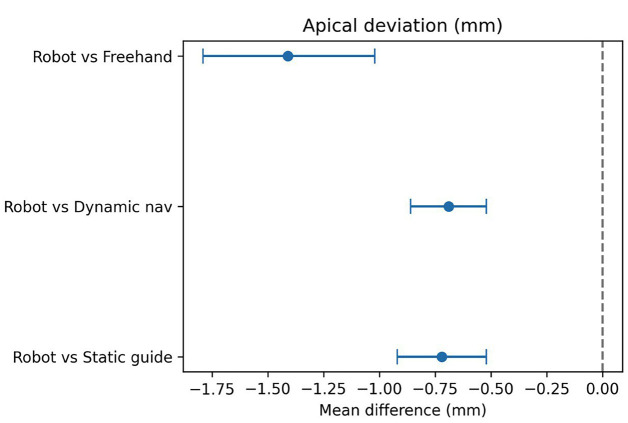
Apical deviation (mm).

**Figure 4:**
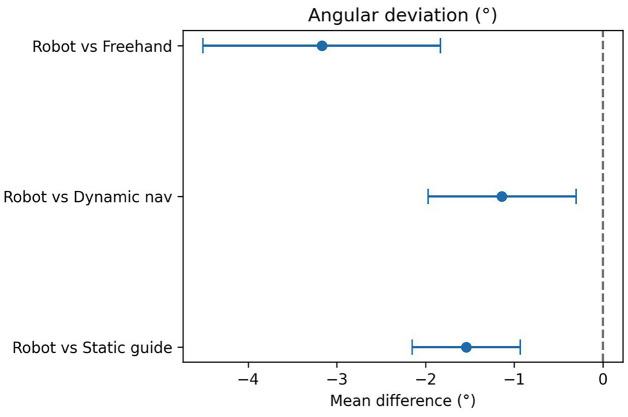
Angular deviation (°).

A total of 18 clinical studies published between 2015 and 2025 were included in the final analysis. These comprised randomized controlled clinical trials (including double-blind designs), as well as prospective and retrospective studies. The studies covered both dental implantation and transoral robotic surgery (TORS) for oropharyngeal tumors. Various types of robotic systems were used, including semi-active installations (RAIS), telemanipulation platforms, and autonomous surgical navigation systems. The primary aim of most studies was to evaluate the clinical efficacy, accuracy, oncological, and functional outcomes of interventions using robotic surgical technology compared with traditional approaches. Characteristics of Interventions and Robotic Systems in Dental Implantation In all studies, robotic methods demonstrated high implant placement accuracy, generally exceeding that of conventional techniques (manual placement, static templates, or dynamic guidance): Linear deviations (coronal and apical) were mostly under 1.0 mm for robotic placement, compared with 1.3-2.1 mm for manual or template-guided approaches. Angular deviations were minimal with robotic interventions, ranging from 1.4° to 3.0°, versus 4°-7° for alternative methods. Several studies (Yang et al., Shi et al., Wang et al., Jia et al., Li et al.) reported statistically significant improvements in all primary accuracy parameters favoring robotic systems (p &lt; 0.001) (12 - 17). Key examples: Yang et al. (2024): semi-automated system achieved 0.76 mm coronal, 0.85 mm apical, and 2.05° angular deviations, outperforming conventional methods (13). Xie et al. (2024): autonomous system showed deviations of 0.53 mm, 0.58 mm, and 1.83° in fully edentulous cases (18). Li et al. (2024) and Zhang et al. (2024): r-CAIS outperformed s-CAIS and d-CAIS across all accuracy parameters (17 , 19). Shi et al. (2025): robotic surgery demonstrated the lowest mesio-distal deviations among RS, DN, and SG approaches (12). Meta-Analysis A random-effects DerSimonian-Laird model was used for quantitative synthesis. Negative mean differences (MD) indicate smaller deviations for robotic methods. Coronal deviation: Robot vs. static template: MD = 0.61 mm (95% CI 1.01 to 0.21 mm; I² = 86%) Robot vs. dynamic navigation: MD = 0.34 mm (95% CI 0.62 to 0.07 mm; I² = 68%) Robot vs. freehand: MD = 1.21 mm (95% CI 1.88 to 0.53 mm; I² = 0%) (Fig. 2) Apical deviation: Robot vs. static template: MD = 0.72 mm (95% CI 0.92 to 0.52 mm; I² = 37%) Robot vs. dynamic navigation: MD = 0.69 mm (95% CI 0.86 to 0.52 mm; I² = 2%) Robot vs. freehand: MD = 1.41 mm (95% CI 1.79 to 1.02 mm; I² = 0%) (Fig. 3) Angular deviation: Robot vs. static template: MD = 1.54° (95% CI 2.15 to 0.93°; I² = 47%) Robot vs. dynamic navigation: MD = 1.14° (95% CI 1.97 to 0.30°; I² = 63%) Robot vs. freehand: MD = 3.17° (95% CI 4.51 to 1.83°; I² = 0%) (Fig. 4) These results demonstrate a statistically significant reduction in linear and angular deviations with robotic implantation compared to alternative methods.

2

3

4

Safety and Clinical Outcomes Across all studies, the safety profile of robotic systems was rated as high. No serious complications related to robotic use were reported. The incidence of intraoperative and postoperative complications was comparable to that observed with conventional techniques. Several studies (Bolding et al., Shi et al., 2024, Wang et al.) specifically highlighted the absence of adverse events during implant placement (14 , 15 , 20). Implant survival was also favorable, with rates reaching up to 100% at 6-12 months, and patient-reported experiences were positive (Nirula et al., 2023) (21). Overall Interpretation The pooled results indicate that robotic surgical systems provide higher accuracy in dental implant positioning compared with conventional methods, particularly in cases of full edentulism and restricted surgical access. Given comparable safety, high predictability, and positive patient reception, robotic systems represent a promising advancement in implantology. Nevertheless, most authors emphasize the need for larger randomized trials with longer follow-up periods to evaluate the cost-effectiveness and long-term stability of outcomes. Kasimoglu et al. (2020) conducted a randomized controlled trial assessing the effectiveness of a humanoid robot in reducing anxiety in children undergoing dental caries treatment. The study included 200 children aged 4-10 years. Anxiety levels were measured using the Fatigue Impact Scale (FIS), heart rate, and observed behavior. The robot-assisted group demonstrated significantly lower anxiety and heart rate (p &lt; 0.05), and 88.3% of children expressed a desire for the robot's presence at future visits, suggesting that robotic technologies can serve as effective behavioral support tools in pediatric dentistry (22). Application of Robotic Systems in Transoral and Reconstructive Surgery Chekkoury Idrissi et al. (2021): Transoral robotic resection of the tongue base in patients with obstructive sleep apnea was safely performed without tracheotomy. In 20 patients, no significant increase in complications was observed, making the procedure less invasive (23). Lin et al. (2022): Compared robot-assisted and conventional contouring surgery of the mandible in 29 patients. The robot-assisted group achieved mean positional deviation of 1.65 mm vs. 2.91 mm, and osteotomy angle of 4.85° vs. 13.26°, demonstrating advantages in both accuracy and safety (24). O'Hara et al. (2024): Secondary analysis of the PATHOS study compared transoral robotic surgery (TORS) and transoral laser surgery (TLS) in 508 patients with HPV-positive oropharyngeal cancer. Four weeks postoperatively, TORS patients had better swallowing function and lower need for tube feeding (7.9% vs. 45%) (25). Larsen et al. (2023): Investigated the impact of dexamethasone dose on pain after TORS. Among 18 patients, no significant differences in pain or other outcomes were found between high and low doses, indicating limited effect of augmented steroid therapy in TORS (26). Theurer et al. (2025, ORATOR study): Assessed swallowing physiology in patients after TORS and radiotherapy (RT). Using the MBSImP scale, no significant differences were observed between groups; however, TORS was associated with transient swallowing impairment at 6 months, highlighting the importance of comprehensive functional outcome evaluation in oropharyngeal surgery (27), (Table 1).

Table 1

## Discussion

This systematic review analyzed 18 clinical studies on the use of robotic systems in implantology, maxillofacial surgery, and transoral oral surgery. The main findings indicate that robot-assisted interventions consistently provide higher accuracy in dental implant placement (linear deviations &lt; 1 mm, angular deviations &lt; 3°) compared with conventional methods (linear deviations 1.3-2.1 mm, angular deviations 4-7°) (12 , 13 , 18). The meta-analysis results support these observations: mean differences for coronal and apical deviations ranged from 0.3 to 1.4 mm, and angular deviations were reduced on average by 1-3° in favor of robotic systems. The largest effect was observed when compared with freehand placement, where linear errors decreased by more than 1 mm and angular errors by 3°. Studies on transoral robotic surgery (TORS) for oropharyngeal tumors showed oncological and functional outcomes comparable to conventional approaches, while improving resection accuracy and preserving swallowing function (23 - 25). The mechanisms underlying the benefits of robotic platforms are well documented. Stable telemanipulation, integrated tracking systems, and three-dimensional visualization reduce the impact of physiological tremor and human error, enhancing accuracy and predictability (30). These features are particularly critical in the confined spaces of the maxillofacial region, especially in premolar and anterior segments, where minimal deviations can compromise functional and aesthetic outcomes. In dental implantology, improved placement accuracy directly reduces the risk of damage to adjacent anatomical structures (mandibular canal, maxillary sinus) and ensures predictable load distribution on implants and surrounding tissues (15 , 31). This can contribute to higher implant survival rates and fewer long-term complications. Studies also reported lower patient anxiety and pain with dynamic navigation, which is important for clinical practice and patient satisfaction (21). Beyond accuracy, several studies assessed functional and patient-reported outcomes. For example, the use of a humanoid robot assistant significantly reduced anxiety and physiological stress responses in children, highlighting the potential for behavioral support in pediatric dentistry (22). In the treatment of obstructive sleep apnea, transoral robotic resection without tracheotomy was shown to be safe (23). Early postoperative swallowing function and quality of life were superior after laser therapy compared with TORS, underscoring the need for individualized technique selection in oncologic practice (25). The safety profile of robot-assisted procedures across all included studies was comparable or superior to conventional methods, with no increase in serious complications or hospital stay duration (14). The review identified several limitations in current research: Heterogeneous study designs (randomized, prospective, and retrospective cohorts), small sample sizes (often 30 patients), and lack of comparable controls for some parameters limit meta-analysis and overall interpretation; Most studies report short-term follow-up (12 months), whereas long-term outcomes such as bone preservation and aesthetic results require extended observation; Economic evaluations are scarce, although high equipment and maintenance costs may restrict access in some clinical settings (32). The learning curve and operative time remain debated. Some authors note longer procedures during initial cases, but with accumulated experience, operative and sterilization times decrease to levels comparable with conventional approaches (33). Training programs, simulation platforms, and standardized robotic protocols can accelerate clinical adoption. Future development of robotic surgery in maxillofacial and dental practice is expected to involve: Integration of artificial intelligence for surgical planning, automatic segmentation of anatomical structures, and complication prediction using big data; Haptic feedback in remote manipulation systems to improve safety and expand the scope of interventions; Multicenter randomized trials with standardized outcome measures, long-term follow-up, and cost-effectiveness analysis, which are essential for establishing clinical guidelines and quality standards (34 , 35).

## Conclusions

Based on the available evidence, robot-assisted technologies in implantology and maxillofacial surgery demonstrate significant potential to improve the accuracy and safety of surgical interventions. However, the overall certainty of evidence, as assessed by the GRADE framework, is moderate to low, warranting cautious interpretation of these findings. To confirm these observed trends, further multicenter studies with larger patient cohorts, extended follow-up, and integrated economic analyses are required. Additionally, the development of training programs and standardized guidelines for clinician education in the use of robotic systems remains an important priority.

## Figures and Tables

**Table 1:** Table Description of individual studies.

Authors and year	Type of research	Patient amount	Research topic	Robotized system	Results	RoB/ROBINS/ research rating
Shi JY et al., 2025 [12]	Randomized controlled clinical research	45	Placement of dental implants in the premolar/molar region	Autonomous robot-assisted system for implant placement	Robotic surgery provided the highest accuracy of implant placement (deviations: platform - 1.1 mm, apex - 1.5 mm, angle - 4.7°). Dynamic navigation and 3D templates showed comparable results (up to 1.9 mm and 5.5°). All methods were associated with favorable healing without complications. Template-based surgeries were shorter and enabled better recovery by day 3; quality of life did not differ between groups.	RoB 2: Low risk of deviation
Fan Yang et al., 2024 [13]	Randomized controlled clinical research	140	Placement of single dental implants	Semi-active robotic system (RAIS)	RAIS demonstrated significantly higher accuracy compared with the freehand method (FHIS): platform deviation - 0.76 mm vs 1.48 mm, apex - 0.85 mm vs 2.14 mm, angular - 2.05° vs 7.36° (p < 0.001). Safety and complication rates were comparable.	RoB 2: Low risk of deviation
Larsen MHH et al., 2023 [26]	Randomized double-blind clinical trial	18	Transoral robot-assisted surgery (TORS)	Transoral robot-assisted surgery (TORS)	Comparison of high and low doses of dexamethasone after TORS revealed no differences in pain intensity (at rest and during swallowing), adverse events, opioid consumption, or other secondary outcomes.	RoB 2: Low risk of deviation
Theurer JA et al., 2025 [27]	Randomized controlled clinical research (ORATOR)	21	Treatment of early-stage oropharyngeal cancer	Transoral robot-assisted surgery (TORS)	The ORATOR sub-study showed no difference in swallowing function between TORS and RT groups according to videofluoroscopy. MBSImP scores increased in both groups, with only a weak correlation to subjective quality of life.	RoB 2: some risk of deviation
Chen JX et al., 2025 [28]	Randomized controlled clinical research	24	Placement of single dental implants	Robotic surgery (RS)	Robot-assisted implant placement provided higher accuracy compared with the freehand method (all p < 0.001): positional and angular deviations were significantly lower. Operating time in the RS group was longer, but complication rates and 6-month implant survival were equally high (100%).	RoB 2: Low risk of deviation
Kasimoglu et al., 2020 [22]	RCCR	200 детей (4–10 лет)	Pediatric dentistry (anxiety reduction)	iRobiQ (humanoid robot)	Reduction in anxiety and pulse in children with robot support; 88.3% of children wanted repeat participation.	RoB 2: some risk of deviation
Li Lin et al., 2022 [24]	RCT	62	Mandibular contouring	Not specified (robotic system for maxillofacial surgery)	High resection accuracy, reduced blood loss, shorter recovery time.	RoB 2: some risk of deviation
Chekkoury Idrissi Y. et al., 2021 [23]	Randomized prospective study	20	Base of tongue resection for OSAS	da Vinci Surgical System	TORS is safe and effective for OSAS; avoiding tracheotomy was associated with fewer complications and reduced operative and hospital time.	RoB 2: High risk of deviation
O'Hara J.T. et al., 2024 [25]	Secondary analysis of a randomized clinical trial (PATHOS sub-study)	508	HPV-associated oropharyngeal carcinoma	da Vinci Surgical System	TORS was associated with a higher rate of nasogastric tube placement (45% vs 8% with TLM) and more pronounced swallowing dysfunction at 4 weeks post-op compared with TLM; no differences in other quality-of-life outcomes were found.	ROBINS-I: Serious risk of deviation
Nirula P. et al., 2023 [21]	Randomized controlled clinical research	60	Placement of dental implants	Navident	The study compared dynamic navigation (DN) and freehand implant placement (FH), evaluating patient satisfaction. DN provided better comfort, less fear, lower pain levels, and higher trust in the procedure. Patients rated the prospects of robotics more positively (4.8 vs 2.0; p < 0.05) and noted reduced operative time (4.63 vs 3.3; p < 0.05).	RoB 2: High risk of deviation
Shi J-Y et al., 2024 [14]	Randomized controlled clinical research	20	Placement of single dental implants	Haptic and machine-vision-controlled collaborative surgery robot	Robot-assisted implant placement provided more precise positioning compared with the manual method: lower deviations in platform (1.23 mm vs 1.9 mm; p = 0.03), apex (1.40 mm vs 2.1 mm; p < 0.01), and angle (3.0° vs 6.7°; p = 0.08). Trauma and complication rates were comparable in both groups.	RoB 2: some risk of deviation
Wang W. et al., 2024 [15]	Preliminary clinical study	13	Placement of dental implants in patients with complete edentulism	Yakebot	Comparison of the Yakebot robotic system and static template (CAIS) showed higher accuracy with Yakebot: lower deviations at the platform (0.65 mm vs 1.37 mm), apex (0.65 mm vs 1.28 mm), depth (0.49 mm vs 0.88 mm), and angle (1.43° vs 3.47°); p < 0.05.	ROBINS-I: Serious risk of deviation
Jia S. et al., 2025 [16]	Retrospective clinical study	39	Placement of dental implants	ADIR	The ADIR robotic system demonstrated significantly higher accuracy compared with static navigation (sCAIS): deviations at the platform (0.43 mm vs 1.31 mm), apex (0.56 mm vs 1.47 mm), and angle (1.48° vs 2.42°); p < 0.001. Accuracy did not depend on implant site. No complications were reported.	ROBINS-I: Serious risk of deviation
Li J. et al., 2024 [17]	Retrospective clinical study	69	Immediate lacement of dental implants in the frontal region	Robotized computer-assisted system (r-CAIS)	The robotic system (r–CAIS) provided the highest accuracy in implant placement: coronal deviation 0.62 mm, apical – 0.65 mm, angular – 1.46°. These values were significantly lower than with s-CAIS and freehand methods (p < 0.05).	ROBINS-I: Serious risk of deviation
Zhang S. et al., 2024 [19]	Retrospective clinical study	77	Placement of dental implants in patients with partial edentulism	r–CAIS and d–CAIS	The autonomous robotic system (r-CAIS) demonstrated significantly higher accuracy compared with dynamic navigation (d-CAIS): lower angular (1.37° vs 4.09°), coronal (0.68 mm vs 1.25 mm), and apical deviations (0.69 mm vs 1.39 mm); p < 0.001. No differences were found in patients subjective VAS ratings.	ROBINS-I: Serious risk of deviation
Xie R. et al., 2024 [18]	Проспективное клиническое исследование	12	Placement of dental implants in cases of complete edentulism	ADIR	During placement of 102 implants with the ADIR system in patients with complete edentulism, high accuracy was achieved: platform deviation — 0.53 mm, apex -0.58 mm, angle - 1.83°. Differences by jaw, site, or other factors were nonsignificant (p > 0.05). Bone and periodontal parameters remained stable over one year.	JBI/NIH: Moderate risk of deviation
Chen W. et al., 2023 [29]	Prospective single-center clinical study	28	Placement of dental implants in patients with partial edentulism	Robotized system	During placement of 31 implants with a robotic system in patients with partial edentulism, high accuracy was achieved: platform and apex deviations -0.53 mm each, angle -2.81°. These values were lower than with static and dynamic CAIS. Differences by jaw, side, and implant size were nonsignificant (p > 0.05).	JBI/NIH: Moderate risk of deviation
Bolding SL, Reebye UN, 2022 [20]	Prospective single-center clinical study	5	Placement of dental implants in cases of complete edentulism	Haptic robotic guidance system	During placement of 38 implants with haptic robotic guidance in patients with complete edentulism, high accuracy was achieved: angular deviation - 2.56°, coronal - 1.04 mm, apical - 0.95 mm, depth - 0.42 mm. No complications were reported.	JBI/NIH: High risk of deviation

1

## Data Availability

The datasets used and/or analyzed during the current study are available from the corresponding author.
